# Patient satisfaction with ExtaviPro™ 30G, a new auto-injector for administering interferon β-1b in multiple sclerosis: results from a real-world, observational EXCHANGE study

**DOI:** 10.1186/s12883-017-0928-9

**Published:** 2017-08-09

**Authors:** Frank A. Hoffmann, Anastasiya Trenova, Miguel A. Llaneza, Johannes Fischer, Giacomo Lus, Dorothea von Bredow, Núria Lara, Elaine Lam, Marlies Van Hoef, Rajesh Bakshi

**Affiliations:** 1 Department of Neurology, Hospital Martha-Maria Halle-Dölau, Halle, Germany; 2 Department of Neurology, Medical University of Plovdiv, Plovdiv, Bulgaria; 3 Neurology Department, Ferrol University Hospital, Ferrol, Spain; 4 Neurologische Praxis (NTDStudy-Group), Lappersdorf, Germany; 5 Multiple Sclerosi Center university of Campania L. Vanvitelli, Naples, Italy; 6 QuintilesIMS, IMS Health GmbH & Co. OHG, Munich, Germany; 7 QuintilesIMS, Barcelona, Spain; 8 Novartis Pharmaceuticals Corporation, East Hanover, NJ USA; 9 Novartis Pharma AG, Fabrikstrasse 12-3.03.12, Postfach, CH-4002 Basel, Switzerland

**Keywords:** ExtaviPro™ auto-injector, Interferon β-1b, Observational, Patient satisfaction, Real-world, Side-effects

## Abstract

**Background:**

Patients with multiple sclerosis (MS) receiving long-term, subcutaneous interferon β-1b (IFN β-1b; Extavia®) often experience injection-site reactions and injection-site pain, which together with other side-effects (such as flu-like symptoms) result in suboptimal treatment compliance/adherence. The EXCHANGE study evaluated patient satisfaction with IFN β-1b treatment, administered using ExtaviPro™ 30G, a new auto-injector, in a real-world setting.

**Methods:**

This 26-week, open-label, prospective, non-interventional, observational, multi-country multi-centre study enrolled patients with MS who had been treated with IFN β-1b or other disease-modifying therapies with a self-administered auto-injector for ≥3 months and who were planned to switch to IFN β-1b treatment administered using ExtaviPro™ 30G as part of routine clinical care. Patient-reported outcomes included overall patient satisfaction (primary outcome) and satisfaction associated with treatment effectiveness, convenience and side-effects, assessed using Treatment Satisfaction Questionnaire for Medication (TSQM)-14. The changes in TSQM scores from baseline to Week 26 were reported. All data were analysed using SAS statistical software (version 9.4).

**Results:**

Of the 336 patients enrolled, 324 were included in the analysis. At baseline, mean ± standard deviation (SD) age of patients was 41.8 ± 11.3 years and 68.2% were women. The mean ± SD of MS disease duration was 6.9 ± 6.6 years, and the majority of patients (94.1%) had relapsing-remitting MS. The mean ± SD of TSQM score for overall patient satisfaction at Week 26 was 75.6 ± 16.46 (baseline, 73.0 ± 17.14; *p* = 0.0342). The mean ± SD of TSQM subscale scores for patient satisfaction with effectiveness, side-effects and convenience were 75.0 ± 18.65 (baseline, 71.6 ± 19.45; *p* = 0.0356), 88.5 ± 18.98 (baseline, 82.7 ± 22.93; *p* = 0.0002) and 77.6 ± 16.72 (baseline, 71.1 ± 17.53; *p* < 0.0001), respectively.

**Conclusion:**

The results from this real-world study suggest that administering IFN β-1b with the new ExtaviPro™ auto-injector significantly improves overall patient satisfaction, including satisfaction associated with effectiveness, side-effects and convenience in MS patients.

## Background

Multiple sclerosis (MS) is a chronic immune-mediated disease characterised by inflammation, axonal damage and demyelination in the central nervous system (CNS) [1]. Patients experience acute transient exacerbations of neurological symptoms (relapses) followed by periods of clinical stabiliosation during the early stages of the disease, and eventually worsening of neurological condition occurs in the form of disability over a period of time [2]. Currently, ~2.5 million people globally have MS [3].

Disease-modifying treatments (DMTs) such as interferons (IFNs; IFN β-1a, IFN β-1b) and glatiramer acetate are commonly prescribed as first-line therapies for relapsing forms of MS [4]. IFN β is postulated to modulate the immune system in MS through several potential pathways. Among these mechanisms, inhibition of T-cell migration from the periphery into the CNS and reduction in metalloproteinase activity on the vascular endothelium that constitutes the blood-brain barrier may be important [5, 6].

IFNs administered subcutaneously or intramuscularly have been shown to reduce the number and severity of clinical exacerbations, disability worsening, need for steroid treatment and the number of hospitalisations in patients with relapsing-remitting MS (RRMS) [7–10]. However, patients receiving long-term subcutaneous IFN β-1b often experience injection-site reactions and injection-site pain/inflammation, together with other side-effects, such as flu-like symptoms [11, 12], resulting in suboptimal treatment compliance and adherence in a substantial number of patients [13, 14].

Injection-site reactions may be related to IFN β-1b being injected or to the injection itself. Use of an auto-injector and thinner needle may improve patient experience and contribute to improving adherence to treatment. Several auto-injectors are available for most IFNs to improve treatment satisfaction, mitigate injection-site reactions and pain concerns associated with mechanical/manual injectors [15–18].

A new, improved auto-injector system, ExtaviPro™ 30G (Novartis Pharma AG), has been developed to facilitate self-administration of IFN β-1b (Extavia®). Compared with previous ExtaviJect® 30G, the new ExtaviPro™ 30G auto-injector is ergonomically designed to make the device easier to use and reliable and to reduce injection-site reactions and pain. The auto-injector facilitates self-administration of high-dose and high-frequency subcutaneous injections of IFN β-1b [19]. ExtaviPro™ uses an ultra-thin 30G needle that reduces shaking or jolting of the auto-injector and facilitates single-handed use and easy access to difficult-to-reach injection sites [19]. Collectively, these features can lead to a better treatment experience and reduced injection-site reactions and associated pain, which can, in turn, improve adherence. ExtaviPro™ 30G is widely available across Europe.

The current non-interventional, real-world study aimed to evaluate patient and nurse satisfaction with ExtaviPro™ 30G auto-injector as a new delivery device for IFN β-1b (Extavia®) in the treatment of MS in the Europe. The primary objective of the study was to evaluate overall patient satisfaction with Extavia® delivered using the new ExtaviPro™ auto-injector. The secondary objectives included patient-perceived effectiveness, convenience and side-effects. Patient tolerability to injection-site reactions, treatment adherence and nurse satisfaction were also evaluated.

## Methods

### Patient population

Men and women aged ≥18 years who had been treated with IFN β-1b (Extavia®) or other injectable first-line DMTs (IFN β-1b [Betaferon®/Betaseron®], IFN β-1a [Avonex®] or glatiramer acetate [Copaxone®]) for MS using a self-administering auto-injector device for at least 3 months before the study entry, who were recommended to switch to IFN β-1b (Extavia®) administered using new ExtaviPro™ 30G auto-injector based on the medical need as part of routine clinical care and in compliance with the local prescribing information, were included in the study. Patients provided written informed consent prior to their enrolment in the study.

### Study design

This was a 26-week, single-arm, open-label, prospective, observational study, conducted between February 2014 and August 2015 at 74 sites in six countries in Europe: Bulgaria, Germany, Greece, Italy, Poland and Spain.

At baseline (Day 1), eligible patients were transitioned to IFN β-1b (Extavia®) self-administered using ExtaviPro™ auto-injector and entered into a 26-week observation period. Because this was an observational study, all patients were eligible for the treatment with IFN β-1b (Extavia®), which was prescribed based on the local prescribing information. Data for the study were collected at routine clinical visits and were recorded by physicians at baseline (Day 1) and at Weeks 4, 13 and 26, except for Bulgaria where data were collected only at Week 26 because routine visits took place every 6 months.

Only patient-reported outcomes were assessed during the study period; no additional laboratory tests or medical procedures were conducted. If required, the dose was titrated in accordance with the locally approved prescribing information.

### Assessments

#### Patient-reported outcomes

##### Treatment satisfaction

The primary endpoint of the study was overall patient satisfaction with IFN β-1b (Extavia®) at Week 26, delivered using the new ExtaviPro™ auto-injector, as assessed by the Treatment Satisfaction Questionnaire for Medication (TSQM). The TSQM (version 1.4) is a 14-item, reliable, validated instrument for assessing patient satisfaction with medication, providing overall scores on four scales including ‘global satisfaction’, ‘effectiveness’, ‘side effects’ and ‘convenience’ [20]. The TSQM was also available in local languages. Patients were asked to refer to their prior treatment and auto-injector for assessment of treatment satisfaction at baseline, and for IFN β-1b (Extavia®) and ExtaviPro™ for all subsequent assessments.

The secondary endpoints of the study included mean scores of individual subscales of the TSQM—‘effectiveness’, ‘side effects’ and ‘convenience’—at Week 26 [20].

#### Tolerability for ExtaviPro™: Pain and injection-site reactions

The tolerability for IFN β-1b (Extavia®) injected through ExtaviPro™ auto-injector consisted of injection-site pain and injection-site reactions at Week 26 and was assessed using the short-form McGill Pain Questionnaire (SF-MPQ) [21] and injection-site reaction questionnaire [22], respectively.

The SF-MPQ comprises 15 descriptors (11 sensory and 4 affective) rated on an intensity scale as follows: 0 = none, 1 = mild, 2 = moderate or 3 = severe. Three pain scores were derived from the sum of the intensity rank values: sensory, affective and total descriptors. Moreover, the SF-MPQ included the Present Pain Intensity Index of the standard MPQ and a visual analogue scale (VAS) [21].

Patients were asked to respond to two questions related to injection-site reactions in the 4 weeks prior to the current visit: ‘Do injection-site reactions occur more or less often now?’ and ‘Do the injection-site reactions cause more or less discomfort now?’ These questions were adapted from the Multiple Sclerosis Treatment Concerns Questionnaire (MSTCQ) [15]. The response was selected from the five options: much less, somewhat less, about the same, somewhat more and much more [22].

#### Adherence

Patient adherence to IFN β-1b (Extavia®) delivered through ExtaviPro™ auto-injector was assessed by the Multiple Sclerosis Treatment Adherence Questionnaire (MSTAQ). The MSTAQ is a 30-item questionnaire designed to identify factors affecting patient adherence and barriers to treatment adherence in patients using MS DMTs. The tool was also designed to predict missed doses. The MSTAQ has three subscales: barriers (score 0–39), side-effects (score 0–40) and coping strategies (score 0–7) [23].

### Healthcare provider-reported outcomes

The proportion of patients reporting incidence of injection-site reactions (specifically pain, swelling, redness, itching or bruising) was evaluated using a physician-completed questionnaire for capturing patients’ reports of reactions over the 4 weeks before the visit [15].

Satisfaction of healthcare providers was evaluated based on nurses’ acceptance of delivering IFN β-1b (Extavia®) through ExtaviPro™ auto-injector, which was assessed using nurse questionnaires. The nurse questionnaires assessed the ease of switching patients to the ExtaviPro™ 30G auto-injector and the overall satisfaction with the injector device (2 items: baseline and follow-up visits). Two questions were asked at baseline visit: ‘How easy was it for the patient to learn to use the device?’ and ‘How easy is it expected to be for the patient to use the device?’ Two similar questions were asked during follow-up visits: ‘Was additional training required?’ and ‘How easy did the patient find it to use the device?’

The response to all questions was selected from the four options: ‘very difficult’, ‘difficult’, ‘easy’ and ‘very easy’. The response to the level of additional training required was selected from the four options: ‘none’, ‘less than half’, ‘more than half’ and ‘repeat entire training’.

### Safety

Safety assessments included reporting of adverse events (AEs) and serious AEs (SAEs).

### Sample size determination and statistical analysis

Assuming a drop-out rate of 15%, 333 patients were planned for enrolment to achieve the target sample size of 283 patients. This sample size allows estimation of the true mean TSQM global satisfaction score within a margin of error of ±2.6 using a two-sided 95% confidence interval (CI) based on an estimated standard deviation (SD) of 22.3 from a historical data.

Treatment satisfaction, tolerability, adherence and other results for the IFN β-1b (Extavia®) group were compared between the previous auto-injector (baseline) and new auto-injector (ExtaviPro™) at Week 26. The change from baseline in the TSQM Global Satisfaction subscale score at Week 26 was analysed using a linear mixed model for repeated measures at 95% CI, with week as the fixed effect and baseline scores as a continuous covariate. A 95% CI was constructed using the least squares mean (i.e. adjusted mean at the overall baseline mean value) and the within-subject variance obtained from the linear mixed model. In case of missing data, the CI was valid under the assumption that the missing data have a missing-at-random mechanism.

Tolerability in terms of satisfaction with the side-effect domain on TSQM subscale, SF-MPQ and the injection-site reaction questionnaires were also analysed using a linear mixed model for repeated measures. The change in MSTAQ scores over time compared with baseline scores was analysed using a linear mixed model for repeated measures following the methodology described above. The treatment compliance rate over time versus baseline scores was analysed using the Chi-square test. Compliance was calculated considering the 2-week period before each follow-up visit, excluding the baseline visit. Given the real-world nature of the data, any missing data were assumed to be missing at random and imputation methods were not applied to ensure description of real patient management in clinical practice.

All data analyses were conducted using SAS statistics software version 9.4 (SAS Institute Inc., Cary, NC) and in accordance with the Strengthening the Reporting of Observational Studies in Epidemiology (STROBE) [24] guidelines and applicable sections of the Consolidated Standards of Reporting Trials (CONSORT) guidelines [25].

### Ethical and good clinical practice

The study protocol and amendment were reviewed and approved by the Independent Ethics Committees and Institutional Review Boards at each centre per local regulations. All patients provided written informed consent before study entry. The study was conducted in compliance with the ethical principles of the Declaration of Helsinki and the International Conference on Harmonisation Good Clinical Practice Guidelines [26].

## Results

### Patient disposition and baseline characteristics

Of the 336 enrolled patients at 74 sites, the majority were receiving IFNβ-1b (93.5%, *n* = 314) and the remaining were receiving other DMTs (6.5%, *n* = 22) as a part of their routine care. In total, 324 patients were included in the final analysis. Twelve patients were excluded from the analysis: one patient (~4.5%) in the other DMTs group did not receive any treatment and 11 (3.5%) in the IFNβ-1b (Extavia®) group used ExtaviPro™ before study inclusion.

Demographics and baseline characteristics of patients are described in Table 1. The overall mean (range) age of patients was 41.8 (19.0–68.0) years, and 68.2% of all patients were women. At baseline, most of the patients (94.1%) had RRMS and the mean duration since diagnosis was 6.9 ± 6.59 years; 41.0% of the patients reported exacerbations during 2 years before study entry, with a mean ± SD of 1.6 ± 0.79 exacerbations per patient. In the IFN β-1b (Extavia®) group, 12.2% of patients had cardiovascular and/or metabolic comorbidities, 9.6% had other neurological disorders, 6.6% had other autoimmune disorders and 4.6% had other inflammatory disorders or osteoporosis.

**Table 1:** Patient demographics and baseline characteristics

	IFN β-1b group *N* = 303	Other DMTs group *N* = 21	Total *N* = 324
Age (years)			
Mean	41.5 ± 11.29	44.9 ± 11.15	41.8 ± 11.30
Median (min–max)	40.0 (19.0–68.0)	43.0 (24.0–68.0)	40.0 (19.0–68.0)
Women, n (%)	207 (68.3)	14 (66.7)	221 (68.2)
Subtype of MS, n (%)			
CIS	7 (2.3)	0 (0.0)	7 (2.2)
RRMS	286 (94.4)	19 (90.5)	305 (94.1)
SPMS	10 (3.3)	2 (9.5)	12 (3.7)
MS disease history			
Duration since MS diagnosis (years)			
Mean	6.7 ± 6.36	9.5 ± 9.16	6.9 ± 6.59
Median (min–max)	4.6 (0.2–35.0)	7.5 (0.3–43.4)	4.8 (0.2–43.4)
Presence of exacerbations, *n* (%)	120 (39.6)	13 (61.9)	133 (41.0)
Number of exacerbations			
Mean	1.5 ± 0.74	1.9 ± 1.14	1.6 ± 0.79
Median (min–max)	1.0 (0.0–5.0)	2.0 (1.0–5.0)	1.0 (0.0–5.0)
Reasons for switching to ExtaviPro™, *n* (%)			
Clinical reasons^a^			
Availability of new auto-injector (patient already treated with Extavia®)	296 (97.7)	2 (9.5)	298 (92.0)
Lack/loss of effectiveness of current treatment, as evidenced by			
returning/worsening/progressing MS symptoms	2 (0.7)	6 (28.6)	8 (2.5)
Increasing frequency of relapses/symptomatic phases	2 (0.7)	3 (14.3)	5 (1.5)
MRI evaluation	3 (1.0)	6 (28.6)	9 (2.8)
Other	5 (1.7)	1 (4.8)	6 (1.9)
Poor tolerance	9 (3.0)	7 (33.3)	16 (4.9)
Patient-centred reasons			
Compliance problem	19 (6.3)	9 (42.9)	28 (8.6)
Patient unable to consistently store current therapy under special conditions	5 (1.7)	2 (9.5)	7 (2.2)

Data are presented as mean ± SD, unless stated otherwise

^a^Multiresponse option

*CIS* clinically isolated syndrome, *DMT* disease-modifying treatment, *IFN* interferon, *max* maximum, *min* minimum, *MRI* magnetic resonance imaging, *MS* multiple sclerosis, *RRMS* relapsing-remitting multiple sclerosis, *SD* standard deviation, *SPMS* secondary progressive multiple sclerosis

At baseline, the majority of patients (94.8%, *n* = 307) were on IFN β-1b (Extavia®). Before switching to ExtaviPro™, 93.5% were using ExtaviJect® auto-injector, 3.4% Avonex® Pen and remaining were on Betaject® Light/Betaject® Lite, Betaject®/Betaject® Comfort/Betacomfort®, Copaxone® autoject 2 or Rebiject II®. The main reasons for switching to ExtaviPro™ auto-injector in the Extavia® group were availability of new injector (97.7%), compliance problems (6.3%) and poor tolerance (3.0%). In the other DMT group, switches were initiated because of compliance problems (42.9%), poor tolerance (33.3%), loss of treatment effectiveness/evidenced by worsening MS symptoms (28.6%), MRI evaluation (28.6%) and increased frequency of relapses/symptomatic phases (14.3%) (Table 1).

### Patient satisfaction with ExtaviPro™

**Fig. 1:**
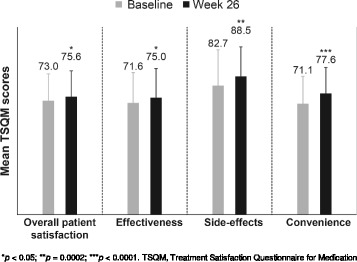
Mean TSQM scores for patient satisfaction at baseline and Week 26. *p < 0.05; **p = 0.0002; ***p < 0.0001. TSQM, Treatment Satisfaction Questionnaire for Medication

Of the 324 included patients, 290 had completed the visit at Week 26. At baseline, 323 patient-reported questionnaires were available and 282 at Week 26. At Week 26, the overall mean ± SD patient satisfaction score on the TSQM scale increased significantly to 75.6 ± 16.46 from baseline (73.0 ± 17.14; *p* = 0.0342, Fig. 1). Patient satisfaction scores on TSQM subscales for effectiveness, side effects and convenience domains at Week 26 were 75.0 ± 18.65 (baseline, 71.6 ± 19.45; *p* = 0.0356), 88.5 ± 18.98 (baseline, 82.7 ± 22.93; *p* = 0.0002) and 77.6 ± 16.72 (baseline, 71.1 ± 17.53; *p* < 0.0001), respectively.

### Tolerability of ExtaviPro™

#### Pain

Tolerability for pain with ExtaviPro™ was assessed using SF-MPQ scores. The mean overall score for total pain of 2.5 ± 4.56 units at Week 26 showed no significant improvement versus baseline (3.3 ± 5.37; *p* = 0.1103) on SF-MPQ.

At Week 26, mean sensory and affective items scores on SF-MPQ were 1.7 ± 3.50 units (baseline 2.3 ± 4.08; *p* = 0.1217) and 0.8 ± 1.46 units (1.0 ± 1.64; *p* = 0.1017), respectively. The mean VAS score of 1.1 ± 1.92 units at Week 26 was not improved significantly versus baseline (1.3 ± 2.03; *p* = 0.3428).

The overall intensity of total pain experience at Week 26 (0.5 ± 0.84) showed no statistically significant improvement versus baseline (0.5 ± 0.87; *p* = 0.6573).

#### Injection-site reactions

**Fig. 2:**
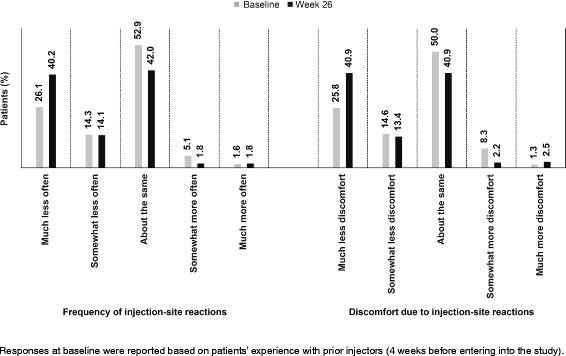
Proportion of patients experiencing injection-site reactions at baseline and Week 26. Responses at baseline were reported based on patients’ experience with prior injectors (4 weeks before entering into the study)

Patient responses to injection-site reactions are presented in Fig. 2. The frequency of injection-site reactions and discomfort at the injection site versus baseline reported as ‘about the same’ decreased by approximately 10% during the study period.

At Week 26, the percentage of patients experiencing less frequent injection-site reactions (combined ‘much less often’ or ‘somewhat less often’) was significantly higher than at baseline (54.3% versus 40.4%; *p* = 0.0006). A significantly higher percentage of patients felt less discomfort due to injection-site reactions at Week 26 (54.3% for combined ‘much less discomfort’ or ‘somewhat less discomfort’) versus baseline (40.4%; *p* = 0.0003).

Responses for incidence of injection-site reactions reported by patients in the MSTCQ questionnaire were in general but not always comparable to responses provided by physicians.

### Adherence to ExtaviPro™

**Fig. 3:**
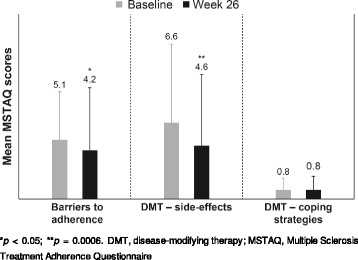
Mean MSTAQ scores for patient treatment adherence at baseline and Week 26. *p < 0.05; **p = 0.0006. DMT, disease-modifying therapy; MSTAQ, Multiple Sclerosis Treatment Adherence Questionnaire

Compared with baseline, a statistically significant improvement was observed for the barriers to adherence score of 4.2 (*p* = 0.0359) and the side-effects score of 4.6 (*p* = 0.0006) at Week 26 on the MSTAQ subscales. Scores in the MSTAQ questionnaire for coping strategies did not change significantly at Week 26 (Fig. 3).

Treatment compliance was achieved when patients had taken at least 80% of medications prescribed at the baseline visit. The percentage of patients who missed an injection during the study period ranged between 13.2% and 22.6%. No statistically significant difference was observed between each study visit versus baseline. At Week 26, a high treatment compliance of at least 96% was reached.

### Nurses’ acceptance for ExtaviPro™

Nurses reported that 94.8% patients found learning to use ExtaviPro™ ‘easy’ or ‘very easy’. They also expected that for the majority (>95%) of patients, future use of the auto-injector would be ‘easy’ or ‘very easy’. The nurses also reported a high level of acceptance of ExtaviPro™ during the study and that the need for additional training to use the device was reduced with time; 98.6% patients needed no additional training to use the new auto-injector at Week 26. Overall, nurses reported that 95.0–97.8% patients felt that the use of the device was ‘easy’ or ‘very easy’.

### Adverse events

Of all reported AEs (*n* = 91), the most frequently reported AEs were headache (34.1%), MS relapse (7.7%), depression (4.4%) and back pain (3.3%); remaining AEs were singular in nature (~1%).

In total, six SAEs were reported, namely hospitalisation due to mild infection, mild neurological decompensation, hospitalisation due to severe vertebroplasty, hospitalisation due to an MS relapse, hospitalisation due to cholecystectomy and death after a cerebral haemorrhage. None of these events were suspected to be related to the study and five of the patients recovered completely. Two women discontinued the treatment due to report of pregnancy during the study.

## Discussion

This non-interventional European study evaluated ‘real-world’ patient-reported outcomes and nurse satisfaction with ExtaviPro™, a newly introduced auto-injector to deliver IFN β-1b (Extavia®) in day-to-day treatment of MS.

Injection-site reactions, pain and other side-effects (such as flu-like symptoms) are common treatment challenges associated with the long-term use of subcutaneous IFN β-1b [11, 12]. This can lead to decreased treatment compliance/adherence in patients with MS [13, 14]. Previous studies have reported that the use of auto-injectors has facilitated self-administration and improved patient compliance to the treatment [17, 18]. ExtaviPro™, an auto-injector featured to have an ergonomic design, single-handed use, an ultra-thin 30G needle and easy access to difficult-to-reach injection sites to reduce injection-site pain on administration of high-dose and high-frequency subcutaneous IFN β-1b.

The patient population in this real-world study included twice as many women as men, with a mean age of approximately 42 years; approximately 94% had RRMS at baseline, which is in line with previously published results on characteristics of MS patients [27, 28]. The majority of patients in this study were already using ExtaviJect® injector (93.5%) for IFN-β1b (Extavia®) administration and the reason for transitioning to the new ExtaviPro™ auto-injector was the availability of the new auto-injector (97.7% patients).

The study evaluated patient satisfaction using the TSQM which is designed to measure treatment satisfaction in chronic diseases [20]. In the recently published TENERE study by Vermersch et al. showed that the TSQM is a useful tool and able to measure psychometric properties in patients with RRMS [29]. In the present study, the overall patient satisfaction score with IFN-β1b (Extavia®) was increased significantly at Week 26. Further, effectiveness, side effects and convenience on the TSQM subscales were also improved significantly. Patient satisfaction for the convenience subscale corroborated with the preference reported in a previous survey [19] in which convenience was rated as an important general attribute for auto-injectors, and patients were more likely to prefer ExtaviPro™ 30G over Betacomfort® auto-injector for routine self-administration of IFN β-1b [19]. Furthermore, the results were in line with the non-interventional EXCELLENT study in which the convenience score with ExtaviJect™ increased significantly between Weeks 6 and 12 on the TSQM subscale [30]. This finding suggests that ExtaviPro™ improved patient treatment experience and satisfaction, particularly in these subscales, and reflects the reliability associated with the use of ExtaviPro™ 30G in MS patients.

In the present study, no significant changes in overall total pain scores, affective items scores, sensory items scores, VAS scores or overall intensity of total pain experience as assessed on the SF-MPQ between baseline and Week 26 were observed. Previous studies reported that the injection-site pain is mainly associated with the needle diameter, and it has been shown that with the use of a thinner needle such as 29G/30G versus 27G, a significantly higher proportion of patients were free from pain throughout the study as assessed on the VAS [15, 31]. In the present study, 93.5% patients were using ExtaviJect® before switching to ExtaviPro™, and the needle size of both the auto-injectors was thin (30G). This could partly explain the lack of a clinically meaningful change in pain scores from baseline to Week 26 as assessed on the SF-MPQ.

No specific questionnaire is available to report responses for injection-site reactions. We adapted the questions from the MSTCQ tool to measure patient satisfaction with injection devices [15]. A considerable percentage of patients (~54%) experienced a lower frequency of injection-site reactions and discomfort due to injection-site reactions at Week 26. This corresponds with the findings reported in a previous study by Mikol and colleagues, in which patients reported significantly lesser injection-site reactions with the auto-injector versus manual injectors (66.1% versus 71.8%; *p* < 0.001) [16].

In the current study, patients reported their experience within 4 weeks, suggesting an improvement using the new auto-injector compared with the previous one. Frequency and discomfort due to injection-site reactions with ExtaviPro™ changed considerably during the study period, reflecting an improvement compared with baseline. Fewer injection-site reactions and discomfort or pain due to injection-site reactions further explains a significant increase in patient satisfaction with ExtaviPro™ 30G on the TSQM subscale for the side-effect subscale.

Adherence to MS therapy as assessed by the MSTAQ did not change significantly between any of the visits with regard to coping strategies. Barriers to adherence and side-effects scores were improved significantly at Week 26 from baseline, suggesting an improvement in patient compliance and fewer side-effects versus baseline. Throughout the study period, a high treatment compliance of at least 96% at all visits was achieved but without statistically significant changes between baseline and Week 26.

Both nurses and patients agreed that ExtaviPro™ was easy to use. Nurses reported that the use of the device had been ‘easy’ or ‘very easy’ to learn and to use for the first time. This result was in line with an earlier international nurse survey in which both patients and nurses perceived that ExtaviPro™ was easy to use and handle [32]. At Week 26, <2% of patients required additional training to use the device, reflecting the user-friendly features of the device. During the study, <5% of patients called or visited a nurse and/or a physician due to concerns related to the use of ExtaviPro™.

No new safety signals were identified during the study. The incidence of the most frequently reported AEs—headache— was 34.1%. None of the six SAEs were suspected to be related to the investigational product. Safety of IFN β-1b administered using ExtaviPro™ was in line with the known safety profile of IFN as reported in earlier studies [7–10].

The study was designed originally to compare the two groups, Extavia® and other DMTs (enrolment in a 1:1 ratio); however, due to the non-interventional nature of the study, only 6.5% of patients were enrolled in the other DMTs group and results were reported using subject-own-control method. Thus, a comparison between the groups and conclusions on the significance of any differences was not possible. Because it was a patient-reported outcome study, a response-recall bias could have occurred as patients would have become familiarised with the questions and study objective over 26 weeks and tended to respond favourably each time.

## Conclusion

In conclusion, this real-world study suggests that administering IFN β-1b through the new ExtaviPro™ auto-injector significantly improves overall patient satisfaction, including satisfaction associated with side-effects, effectiveness and convenience on the TSQM subscales in MS patients. ExtaviPro™ was easy to use, contributed to patient satisfaction and was well tolerated, which may support better adherence to the treatment.

## Abbreviations

AE: Adverse event
CI: Confidence interval
CIS: Clinically isolated syndrome
CNS: Central nervous system
CONSORT: Consolidated Standards of Reporting Trials
DMT: Disease-modifying treatment
IFN: Interferon
Max: Maximum
Min: Minimum
MRI: Magnetic resonance imaging
MS: Multiple sclerosis
MSTAQ: Multiple Sclerosis Treatment Adherence Questionnaire
MSTCQ: Multiple Sclerosis Treatment Concerns Questionnaire
NSAIDs: Non-steroidal anti-inflammatory drugs
RRMS: Relapsing-remitting multiple sclerosis
SAE: Serious adverse event
SD: Standard deviation
SF-MPQ: Short-form McGill Pain Questionnaire
SPMS: Secondary progressive multiple sclerosis
STROBE: Strengthening the Reporting of Observational Studies in Epidemiology
TSQM: Treatment Satisfaction Questionnaire for Medication
